# High Myopia Prevalence across Racial Groups in the United States: A Systematic Scoping Review

**DOI:** 10.3390/jcm12083045

**Published:** 2023-04-21

**Authors:** Bryana Banashefski, Michelle K. Rhee, Gareth M. C. Lema

**Affiliations:** 1 Department of Ophthalmology, Icahn School of Medicine at Mount Sinai, New York, NY 10029, USA; 2 John J. Peters VA Medical Center, Bronx, NY 10468, USA

**Keywords:** high myopia, visual impairment, scoping review, underrepresented

## Abstract

High myopia is a significant public health issue globally and in the United States (US), where it affects ~4% of the population or 13 million people. This is a potentially blinding condition, but complications can be prevented with early intervention in childhood. Several countries have developed robust data on high myopia, but the United States' data on high myopia remains lacking. Further, underrepresented populations are at particular risk of complications due to reduced access to optometric and ophthalmic care. We performed a systematic scoping review of population-based studies that investigated the prevalence of high myopia across racial and ethnic groups in the US to identify the impact of high myopia on underrepresented communities. Only four studies were identified that met inclusion criteria, which highlights the need to further investigate the topic in the United States. The prevalence of high myopia ranged from a low of 1.8% among Hispanic populations to a high of 11.8% among Chinese populations. Our study demonstrated a paucity of high myopia data in the United States and variable rates of high myopia depending on the time and location of each study. More complete prevalence data will help identify opportunities for community-based interventions to prevent debilitating and blinding complications of high myopia.

## 1. Introduction

Myopia is the leading cause of visual impairment globally, even reaching epidemic levels in the most highly affected countries [1]. High myopia is the most severe form and can result in blinding complications. Unrecognized and untreated myopia presents a risk of developing into high myopia. High myopia is characterized by potentially severe anatomic changes in the retina that can limit best corrected visual acuity [2]. It has been estimated that globally, the number of people with vision loss resulting from high myopia could increase seven-fold from 2000 to 2050, and myopia would become a leading cause of permanent blindness worldwide [3]. Axial length elongation has been found to be related to the severity of refractive error and, subsequently, high myopia [4]. Complications such as glaucoma, retinal detachment, and myopic maculopathy can cause irreversible blindness [5].

Although the impact of high myopia in at-risk regions has been robustly studied internationally [6], population-based studies of high myopia in the United States (US) are more limited in number, highlighting a lack of robust understanding of high myopia in this country. In the United States, the latest estimates show myopia’s prevalence to be 41.6% [7]. In a study analyzing the change in the prevalence of myopia over 30 years, the United States showed a 66% increase [7]. Today, high myopia impacts almost 4% of the United States population, which is equivalent to ~13 million people [8]. 

The window of opportunity for high myopia prevention, or myopia control, is during childhood and requires access to eye screening and appropriate treatment. Being a member of a minority racial group and/or having lower socioeconomic status creates barriers to accessing primary eye and vision care [9,10]. Further, racial minorities are disproportionately affected by poverty and lower socioeconomic status in the United States [11].

Given the importance of early intervention to curb the progression of myopia into high myopia, the varying rates of myopia prevalence and growth across racial groups in the United States, and the relatively affordable public health interventions available for early myopia intervention, it is important that we understand the racial influence on eye care access in the United States. To date, no systematic scoping reviews exist on high myopia prevalence across racial groups in the United States. This review will summarize the current body of literature on high myopia prevalence across racial groups in the United States and discuss the implications of the available data.

## 2. Materials and Methods

This scoping review followed the framework of the Preferred Reporting Items for Systematic Reviews and Meta-Analyses extension for Scoping Reviews (PRISMA-ScR). See checklist in Supplementary Material (Supplementary File S1).

### 2.1. Search Strategy and Quality Assessment

A systematic search to identify papers on high myopia prevalence across racial groups in the US was conducted on 15 July 2022 in consultation with an experienced Medical Research Librarian. We collaborated with the Medical Research Librarian to develop search term queries and keywords, and to define our search strategy. Our search included the following databases: Ovid Embase, Ovid MEDLINE, and Web of Science Core Collection. The search was restricted to articles available online. The searches were designed to query each database using unique subject headings and consistent keywords (further details on the search strategy, subject headings, and keywords are available in Supplementary Material; Supplementary File S2). No limits were placed on publication date. We also examined reference lists for additional relevant articles. The quality of the search was evaluated by confirming all known articles on the topic that appeared in the search. 

An export from each database was imported into EndNote Library on 15 July 2022. After Endnote automatic deduplication, several duplicates remained, requiring manual deduplication. One author (BB) manually deduplicated using article title, author names, and year of publication. Due to limitations of search functionality across queried databases, excluding non-US studies within the search was not possible. Given our review’s focus on a US population, one reviewer (BB) manually reviewed each article title and abstract and removed articles that did not include a US population. After removing non-US studies, articles were imported into Covidence (Covidence Systematic Software, Veritas Health Innovation, Melbourne, Australia) on 18 July 2022. 

Two authors (BB and GL) independently screened the titles and abstracts to exclude articles that were not eligible. Discrepancies between the two reviewers were resolved by team consensus. Two authors (BB and GL) independently appraised the full text of the identified papers. Discrepancies between the two reviewers were resolved by team consensus. 

### 2.2. Inclusion and Exclusion Criteria

We searched for population-level studies that described the prevalence of high myopia across racial groups in the United States. Exclusion criteria included abstracts, non-US population, lack of prevalence data on high myopia, lack of prevalence data across racial groups, and studies that included prevalence for a non-population-level patient cohort (e.g., high myopia prevalence among cohort of children with red–green color blindness).

As of 2019, the standardized definition of high myopia is ≤−6.00 D, based on a published consensus and its use in a majority of interventional studies [12]. Historically, the definition of high myopia varied within the literature, including: ≤−5.00 diopters (D), ≤−6.00 D, or ≤−8.00 D [13,14,15,16]. Given the historic variation and the timeline of this review including dates prior to the established standard, we did not exclude papers that used a definition of high myopia other than the currently accepted definition of ≤−6.00 D. 

### 2.3. Assessment of Study Quality

We assessed study quality with the appraisal tool to assess the quality of cross-sectional studies (AXIS) [17]. 

### 2.4. Data Extraction and Synthesis

We extracted high myopia prevalence data across racial groups from the included articles. If prevalence was reported for an age range, we calculated a weighted average based on the number of subjects per age range and prevalence data per age range. If prevalence was reported for male and female individuals separately, we calculated the weighted mean value of both groups to represent prevalence in our paper. 

## 3. Results

**Figure 1:**
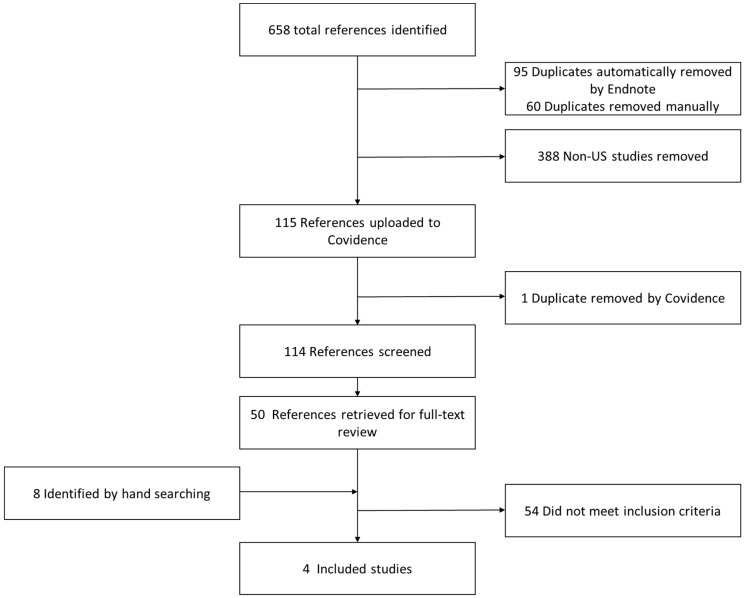
Process for selecting articles.

Our process for selecting articles is shown in Figure 1. The search strategies identified 658 papers. After deduplication, 503 articles remained. After the removal of non-US studies, 115 articles remained. After abstracts were read for eligibility, 50 articles were retained to be read completely; 8 more were identified by reviewing reference lists. Of these 58, 4 articles met the inclusion criteria. The final sample used for data extraction was thus four articles.

### Data Synthesis

The Baltimore Eye Study (BES) [14] presented data across an age range. Weighted averages were used to determine high myopia prevalence. The remaining studies presented data as seen in Table 1.

The four studies included in this review have a total of 19,267 eyes, using 1 eye per participant. The CHES study found high myopia prevalence in the Chinese racial group to be 8.6%. The MESA study found high myopia prevalence in the Black, Chinese, Hispanic, and White racial groups to be 3.1%, 11.8%, 1.8%, and 5.4%, respectively. The LALES study found high myopia prevalence in the Hispanic racial group to be 2.4%. The BES study found high myopia prevalence in the Black and White racial groups to be 0.9% and 1.8%, respectively. 

Three out of the four studies used the worse eye for analysis, defined as the eye with the larger absolute spherical equivalent (CHES [15], MESA [18], LALES [19]). One study (BES [14]) used the right eye for analysis.

Methods to capture refractive error varied slightly across the four studies. All four studies used noncycloplegic refraction. Two out of the four studies (CHES and LALES) used autorefraction plus subjective refinement. BES used manifest refraction only. MESA used autorefraction only. 

Population survey methods differed across the four studies. Two studies (CHES and LALES) used census tracts to define sampling frames in specific geographic areas (ten census tracts in Monterey Park, California, and six census tracts in La Puente, California, respectively). BES used a cluster sample survey methodology of 16 clusters across Baltimore, Maryland. BES clusters were stratified to include equal proportions of Black and White adults. The population-based sample for MESA was created from site recruitment across geographic areas in the US (Forsyth County, NC, Northern Manhattan and the Bronx, NY, Baltimore City and Baltimore County, MD, St. Paul, MN, Chicago, IL, Los Angeles County, CA). Notably, the MESA database includes adults who are free of clinical cardiovascular disease at the time of recruitment. CHES, LALES, and BES used at-home, in-person interviewing techniques to screen for eligibility and recruit participants. MESA used mailed brochures and telephone calls for recruitment. 

BES defined high myopia at ≤−6.0 D, which aligns with the current, standardized definition of high myopia [12]. The CHES, LALES, and MESA studies define high myopia more liberally at ≤−5.0 D.

Study quality was assessed in all publications using the Appraisal tool for Cross-Sectional Studies (AXIS) [17]. The AXIS assesses the quality of cross-sectional studies based on the following criteria: clarity of aims/objectives and target population; appropriate study design and sampling framework; justification for the sample size; measures taken to address non-responders and the potential for response bias; risk factors/outcome variables measured in the study; clarity of methods and statistical approach; appropriate result presentation, including internal consistency; justified discussion points and conclusion; discussion of limitations; and identification of ethical approval and any conflicts of interest. The scoring system uses a “yes,” “no,” or “do not know/comment” design. Review articles were categorized into quartiles: >15 AXIS, criteria met, 10-15 AXIS criteria met, 5-9 AXIS criteria met, and ≤4 AXIS criteria met. All four articles met >15 AXIS criteria (See Supplementary File S3). 

## 4. Discussion

This review highlights three key findings which will be discussed below: (1) the overall lack of literature on high myopia prevalence across racial groups in the US, (2) the high degree of variation of high myopia prevalence across racial groups in the US, and (3) the prevalence of high myopia is a significant public health problem. 

### 4.1. The Paucity of Data and High Degree of Variability

Only four studies include high myopia prevalence data among the general populations across racial groups in the US. This highlights a lack of understanding among the ophthalmology community on the true prevalence of this disease in the US. Among these four articles, prevalence data for the same racial groups differed across studies. 

There are several reasons for this discrepancy. It can be due to the time that passed in between studies. For example, comparing findings in BES to Black and White racial groups in MESA, 19 years had passed when comparing the dates that data were collected. During that period, the incidence of myopia increased among all groups worldwide [13].

Additionally, BES used the currently accepted standardized definition for high myopia at ≤−6.00 D as compared to the three other studies (CHES, LALES, MESA) which used a more liberal definition for high myopia at ≤−5.00 D. Flitcroft et al. (2019) established consensus criteria based on ≤−6.00 D being used in a majority of interventional studies. This definition has become a generally agreed upon criterion, including among the American Academy of Ophthalmology (AAO), the American Association for Pediatric Ophthalmology and Strabismus (AAPOS), and the International Myopia Institute (IMI) [20,21,22]. All four studies were published prior to 2019 when a standardized definition for high myopia was established at ≤−6.00 D [12]. Differing criteria for high myopia complicates the analysis of results across studies. Given this difference in definition, the CHES, LALES, and MESA findings could overrepresent high myopia prevalence overall. 

It should be noted that the risks of myopic degeneration and nonrefractive complications are more likely at greater levels of myopia [23]. Therefore, assessing high myopia from a refractive standpoint makes sense when trying to understand the societal impact of visual deficits created by myopia, but may have less significance when counseling myopic individuals on their risk of nonrefractive complications.

The CHES and MESA study concluded different prevalence percentages for the Chinese racial group (8.6% and 11.8%, respectively). The MESA study included patients without cardiovascular disease at the time of recruitment which could have introduced selection bias and reduced the sample size of subjects with high myopia [18]. 

Comparing LALES findings to the Hispanic racial group in MESA (2.4% to 1.8%, respectively), we see the LALES study found a higher prevalence. It is important to note the difference in terminology used across these two studies. LALES study used the term “self-identified Latino ethnicity.” LALES further broke down their patient population by ancestry finding 95% of patients self-identified as Mexican-American ancestry and 5% self-identified as Native American ancestry. The MESA study used the term “Hispanic” and did not provide a further breakdown of their self-identified racial groups. 

Regardless of the variation between studies, the findings highlight a high degree of variability among high myopia prevalence between racial groups. For example, if we look within the MESA study alone, prevalence ranges from 1.8% in the self-identified Hispanic population to 11.8% in the self-identified Chinese population. Even though the CHES study found a lower prevalence percentage for the Chinese racial group when compared to MESA at (11.8% compared to 8.6%, respectively), the CHES prevalence finding for the Chinese racial group is still several percentage points higher than the next closest prevalence percentage across all studies. This underscores the well-established finding in the literature that populations of Asian descent have a higher prevalence of high myopia [13]. 

Based on these data, we can conclude that there is variation among high myopia prevalence across racial groups in the US. Large-scale population-based studies may improve our estimates of high myopia prevalence in the United States. 

### 4.2. The Public Health Problem

Although the prevalence of high myopia appears relatively uncommon among racial groups, the population affected is large for a blinding and debilitating ophthalmic disease. We can estimate the national impact of high myopia by applying prevalence data to national populations of each race or ethnic group. Data from the 2020 decennial census [24] revealed Asian, Black, Hispanic, and White populations of, respectively: 20 million, 41 million, 62 million, and 204 million. The Chinese population was estimated at 5.1 million in the 2018 population survey [25]. 

Using the data presented here, the impact of high myopia upon underrepresented minorities would have a low estimate of 1.8 million people (370,000 Black and 1.1 million Hispanic) and a high estimate of 2.8 million people (1.3 million Black and 1.5 million Hispanic). 

The low and high impact on the Chinese community would be 438,000 and 601,800, respectively. Since the Chinese prevalence data reflects higher rates of myopia among all Asian populations [13], and the Chinese population is only one-quarter of all Asians in the US [26], the true impact is likely much greater, even though it cannot accurately be calculated with the data in this study. 

Including all populations here, the public health impact is even more significant. The low and high estimates of country-wide prevalence would be 5.6–14.4 million people across all groups. This was corroborated in a large study of myopia in children by Theophanous, et al. (2018) in which the prevalence of myopia ≤ 5.0 D reached 9.2% in 17–19-year-olds, the oldest age group [27].

Racial groups with limited access to preventative eye care are at risk of allowing myopia to progress unnecessarily. Studies have shown a difference in access to eye services across racial groups in the United States. Kemper et al. found that for Medicaid-enrolled children in urban counties, Black or Hispanic children had lower odds of receiving care from an optometrist or ophthalmologist than non-Hispanic/non-Black children, regardless of age, gender, family income, or health insurance status [28]. A 2009 National Eye Institute-sponsored study based in Los Angeles County found that there is a substantial degree of unmet need for refractive correction among Hispanic and Black children that can be corrected [29]. 

In 2009, Vitale et al. analyzed the change in the prevalence of myopia in the United States over 30 years by comparing myopia prevalence in 1999–2004 to myopia prevalence in 1971–1972 [7]. Overall, the prevalence of myopia was 66.4% (41.6% vs. 25.0%) higher among participants aged 11–54 years in the 1999–2004 cohort. The analysis looked at race, and the difference in prevalence was greater for Black participants than for White participants. Black participants’ myopia prevalence more than doubled in 1999–2004 compared to 1971–1972.

The results of this review emphasize the potentially high impact of focusing efforts to curb myopia progression. Targeting racial groups that have a larger degree of risk for high myopia could generate the greatest benefit, whether that is for a high degree of prevalence (i.e., Chinese racial group) or for a historic lack of equal access to preventative eye care (Black and Hispanic groups).

### 4.3. Myopia’s Impact on Education and Learning

A disease that can limit best-corrected vision can have a significantly negative impact over a lifetime. Vision impairment can have substantial negative social and economic consequences including loss of productivity, low academic performance, and diminished quality of life [30,31]. Vision disorders are among the most prevalent handicapping conditions in children in the United States [32]. For children, vision impairment’s impact on learning is well established. If a child struggles with their vision, they may not be able to succeed in school [33]. 

Studies have found that children with vision impairment make more reading errors when reading small print and more errors on spelling tests than students with normal vision [34,35]. Other studies showed significantly lower achievement scores and literacy skills among first through fifth and eighth graders with uncorrected hyperopia [36,37]. Correcting vision can improve academic performance; a 2021 study found that a school-based vision program that provided eyeglasses to students increased performance on the i-Ready reading test and i-Ready mathematics test, which is a standardized test given to Maryland students in grades 1–8 [38]. 

Given the established connection between vision and learning and the variation in access to eye care in the United States, we must consider the long-term negative impact on individual quality of life when high myopia goes undetected and/or untreated and the role public health programs can play in identifying and treating myopia early in life for populations that are most vulnerable. 

### 4.4. Treatment for Myopia Control

Genetics and environmental factors both have been shown to influence myopia. Treatment options to curb myopia progression include optical correction, pharmacologic intervention, and environmental and behavioral modifications. Interventions to curb myopia progression have focused on modifiable environmental factors. Outdoor time has been shown to be a protective factor for myopia [39]. A meta-analysis by Recko et al. found 2% reduced odds of myopia for each additional hour of time spent outdoors per week. This offers a low-cost, tangible intervention for reducing myopia risk [39]. 

Near work (i.e., reading or screen time) has also been targeted as a modifiable behavioral factor that could influence myopia, however, a systematic review by Lanca et al., 2020, found mixed results for near work as a risk factor for myopia and called for more objective screen time measures for future studies [40].

For optical correction, myopia progression can be managed with the use of multifocal contact lenses [41,42], orthokeratology [43,44], or spectacles [45,46]. Spectacles currently have the weakest evidence, although a multicenter clinical trial is ongoing in the United States (NCT03623074). 

For pharmacologic intervention, treatment modalities include the following muscarinic receptor antagonists: topical atropine and pirenzepine [47,48,49,50]. However, appropriate concentrations of atropine and pirenzepine are not commercially available in the United States. They must be compounded at specialty pharmacies, which reduces access and can increase out-of-pocket costs. 

There are several ongoing trials for low-concentration topical atropine in Europe and the United States [2]. Phase 3 trials are actively recruiting; the Myopia Treatment Study is planned to be completed in November 2022 (NCT03334253). If shown to be beneficial, these products could improve access to reasonable pharmacologic interventions. 

Fricke et al. (2022) compared the cost of implementing active myopia control to traditional myopia management in China and Australia and found that active myopia control (including contact lenses, spectacles, and atropine drops) initiated at a young age reduced overall lifetime costs compared to traditional myopia management [51]. Traditional myopia management provided single-vision optical correction of a person’s full refractive error throughout childhood and beyond, responding to progression and complications as they occur [51]. Cost-saving endpoints included: reduced refractive progression, simpler corrective lenses, fewer lens replacements, reduced risk of eye disease and vision loss, and reduced management of myopia complications. 

The treatment of myopia is multimodal and includes relatively affordable components. There is an opportunity for myopia treatment programs to be implemented at the state or national level to address the growing disease burden of high myopia. Organizations developing programs would be able to decide the appropriate scale of their myopia treatment programs based on resourcing and the needs of their community. For example, one program might offer public health campaigns prompting behavioral modifications only while another might also fund multifocal contact lenses and atropine. 

### 4.5. Curb Myopia Progression through Public Health Initiatives

Myopia progression and high myopia are strategically positioned for public health intervention. Given that the window of opportunity for intervention is in childhood and that early intervention reduces lifetime costs [51], it is both beneficial to patients and fiscally responsible to develop programs that identify and treat myopia progression in the United States. The American Academy of Ophthalmology (AAO) created the Task Force on Myopia in 2019 and is working on multidisciplinary problem-solving strategies that incorporate school-based programming and education for pediatricians and family physicians [52]. 

Vision screening for school children is not currently mandated at the federal level in the United States; it is managed at the state level. As of 2021, 41 states require vision screening for school children [53]. In a 2021 study evaluating vision screening requirements at the state level, Wahl et al. found significant variation in vision screening methods and vision screening timing across state regulations. Standardization of vision screening protocols across all states that are anchored in evidence-based medicine would be an effective first step in identifying and treating preventable myopia progression. Additionally, public health programs that cover the cost of care for myopia progression for high-risk patients would be mutually beneficial: helping reduce patients’ lifetime symptoms (i.e., reducing the risk of blindness) and reducing overall healthcare costs.

Proactive programs can be implemented to help reduce the risk of myopia progression. Programs can be implemented to encourage more outdoor time, particularly in young school children [39]. For example, the Ministry of Education in Taiwan implemented an intervention that had schools take school children outside for 120 min a day. A prospective analysis of this intervention found that when children spent more time outdoors, the rate of myopia progression to low vision decreased [50].

Additionally, China limits video game screen time for children under 18 years old to 3 h a week [50,54], and Singapore initiated a public health campaign to encourage children to play outside [55]. Cost-effective programs that have successfully been implemented in other countries to combat the rise in myopia progression can inspire effective public health strategies to address high myopia in the United States.

## 5. Limitations

The number of studies that met the criteria for inclusion was small, which limits our ability to generalize the findings. Nonetheless, we identified and addressed several points of discussion that may lead to areas of future research. This study was limited in scope to the United States, which allowed us to focus our discussion on the implications of our findings and approaches to prevent the progression of high myopia. In addition, the definition of high myopia differed across the studies included in our analysis. Three studies used a more liberal definition of high myopia compared to today’s standardized definition, potentially overrepresenting the prevalence of high myopia. The age ranges varied across the four studies, which could impact our results as myopia is a progressive disease and might be more prevalent in older populations. 

## 6. Conclusions

High myopia is a devastating disease that negatively impacts quality of life. We present the first systematic scoping review of high myopia prevalence among underserved populations in the United States. We observed a paucity of population-based studies that could be used to accurately estimate differences among racial and ethnic groups. Additionally, differences in methodology, such as varied definitions of high myopia, further complicated comparisons across studies. 

Our findings have established the need for greater efforts to understand the prevalence of high myopia among diverse populations within the United States. 

High myopia may be preventable if confronted during childhood. The international literature has demonstrated effective behavioral modifications and interventions to prevent myopia progression. A better understanding of high myopia’s impact across racial groups in the United States can help inform targeted, effective public health initiatives that can curb myopia progression and preventable blindness while decreasing overall healthcare costs that span a lifetime.

## Figures and Tables

**Table 1:** Prevalence of high myopia across racial groups in the United States.

Authors	Dataset/ Study	Number of Participants	Study Publication Year	Data Collection Year	Study Population	Racial Groups	High Myopia Definition	High Myopia Prevalence
Varma, et al. [15]	The Chinese American Eye Study (CHES)	4582 eyes	2016	2009–2013	Residents in ten census tracts in Monterey Park, California Age 50 or older	Self-identified Chinese Americans	≤−5.0D	Chinese—8.60%
Pan, et al. [18]	Multi-Ethnic Study of Atherosclerosis (MESA)	4430 eyes	2013	2002–2004	Prospective cohort study sampled from: Baltimore, Maryland; Chicago, Illinois; Forsyth County, North Carolina; Los Angeles, California; New York, New York; and St Paul, Minnesota restricted to adults without known cardiovascular disease Age 45–84 years old	Self-identified: Black—1230 (27.8%) Chinese—487 (11%) Hispanic—1046 (23.6%) White—1667 (37.6%)	≤−5.0D	Black—3.1% Chinese—11.8% Hispanic—1.8% White—5.4%
Tarczy-Hornoch, et al. [19]	Los Angeles Latino Eye Study (LALES)	5396 eyes	2006	2000–2003	Residents in six census tracts in La Puente, California Age 40 or older	Self-identified Latino ethnicity	≤−5.0D	Hispanic—2.40%
Katz, et al. [14]	Baltimore Eye Survey (BES)	4859 eyes	1996	1985–1988	Population- based sample survey in Baltimore Age 40 or older	Self-identified: Black—2200 (45%) White—2659 (55%)	≤−6.0D	Black—0.9% White—1.8%

## Data Availability

Original data presented in this study are available in references [11,12,13,14].

## Supplementary Materials

The following supporting information can be downloaded at: https://www.mdpi.com/article/10.3390/jcm12083045/s1, Supplementary File S1: PRISMA-ScR Checklist [56], Supplementary File S2: Search Strategy; Supplementary File S3: the Appraisal tool for Cross-Sectional Studies Checklist.
