# Supplementary material for: High Myopia Prevalence across Racial Groups in the United States: A Systematic Scoping Review
Source: J Clin Med. 2023 Apr 21;12(8):3045. doi: 10.3390/jcm12083045 (PMC10144975; doi:10.3390/jcm12083045)
Supplement: Supplementary file 1 [file jcm-12-03045-s001.zip › Supplementary File S2_Search Strategy Terms.pdf]

Supplementary Material S2. Search Strategy Terms

Ovid MEDLINE(R) and Epub Ahead of Print, In-Process, In-Data-Review & Other Non-Indexed Citations and Daily <1946 to July 14, 2022>

1	exp racial groups/	242245
2	african American*.mp.	92938
3	african ancestry.mp.	2238
4	Asian*.mp.	168560
5	black*.mp.	201083
6	Caucasian*.mp.	67580
7	Hispanic*.mp.	71537
8	Indian*.mp.	114252
9	latino*.mp.	40118
10	Latina*.mp.	5100
11	minority.mp.	81242
12	native American*.mp.	5995
13	indigenous.mp.	41243
14	pacific islander*.mp.	15458
15	people of color*.mp.	1202
16	white*.mp.	450929
17	(racial or race).mp.	174472
18	1 or 2 or 3 or 4 or 5 or 6 or 7 or 8 or 9 or 10 or 11 or 12 or 13 or 14 or 15 or 16 or 17	1117178
19	prevalence.mp. or prevalence/	825942
20	epidemiology/ or epidemiology.mp.	2099101
21	exp United States/ep [Epidemiology]	229377
22	19 or 20 or 21	2469687
23	high myopia.mp.	4384
24	18 and 22 and 23	171

Embase Classic+Embase <1947 to 2022 July 14>

1	exp ancestry group/	377182
2	african American*.mp.	131075
3	african ancestry.mp.	3548
4	Asian*.mp.	217609
5	black*.mp.	342244
6	Caucasian*.mp.	196055
7	Hispanic*.mp.	117338
8	Indian*.mp.	157022
9	latino*.mp.	21205

10 Latina*.mp. 6395
11 minority.mp. 111711
12 native American*.mp. 8455
13 indigenous.mp. 51752
14 pacific islander*.mp. 8845
15 people of color*.mp. 1396
16 white*.mp. 607875
17 (racial or race).mp. 305098
18 1 or 2 or 3 or 4 or 5 or 6 or 7 or 8 or 9 or 10 or 11 or 12 or 13 or 14 or 15 or 16 or 17 1728817
19 prevalence.mp. or prevalence/ 1270084
20 epidemiology/ or epidemiology.mp. 1591635
21 exp epidemiological data/ 4245031
22 19 or 20 or 21 5372414
23 high myopia.mp. or exp high myopia/ 7098
24 18 and 22 and 23 148

Web of Science Core Collection- 339 results

TS=(african American OR african ancestry OR Asian OR black* OR Caucasian* OR Hispanic* OR Indian* OR latino* OR Latina* OR minority OR native American* OR indigenous OR pacific islander* OR people of color* OR white*)
OR
TS=(racial or race)

AND

TS=(prevalence OR epidemiology epidemiological)

AND

TS=(high myopia)
