# Supplementary material for: High Myopia Prevalence across Racial Groups in the United States: A Systematic Scoping Review
Source: J Clin Med. 2023 Apr 21;12(8):3045. doi: 10.3390/jcm12083045 (PMC10144975; doi:10.3390/jcm12083045)
Supplement: Supplementary file 1 [file jcm-12-03045-s001.zip › Supplementary File S3_Risk of Bias Assessment.pdf]

AXIS Tool: Risk of bias assessment

Note: This is AXIS tool developed for a critical assessment of the quality of cross-sectional studies [1]

Abbreviations: ND – not described; NS – not stated

	**The Chinese American Eye Study (CHES) (2016)**	**Multi-Ethnic Study of Atherosclerosis (MESA) (2013)**	**Los Angeles Latino Eye Study (LALES) (2006)**	**Baltimore Eye Survey (BES) (1996)**
1. Were the aims/objectives of the study clear?	Yes	Yes	Yes	Yes
2. Was the study design appropriate for the stated aim(s)?	Yes	Yes	Yes	Yes
3. Was the sample size justified?	Yes	Yes	No	No
4. Was the target/reference population clearly defined? (Is it clear who the research was about?)	Yes	Yes	Yes	Yes
5. Was the sample frame taken from an appropriate population base so that it closely represented the target/reference population under investigation?	Yes	Yes	Yes	Yes
6. Was the selection process likely to select subjects/participants that were representative of the target/reference population under investigation?	Yes	Yes	Yes	Yes
7. Were measures undertaken to address and categorize non-responders?	No	No	No	No
8. Were the risk factor and outcome variables measured appropriate to the aims of the study?	Yes	Yes	Yes	Yes
9. Were the risk factor and outcome variables measured correctly using instruments/ measurements that had been trialled, piloted or published previously?	Yes	Yes	Yes	Yes
10. Is it clear what was used to determined statistical significance and/or precision estimates? (e.g., p values, CIs)	Yes	Yes	Yes	Yes
11. Were the methods (including statistical methods) sufficiently described to enable them to be repeated?	Yes	Yes	Yes	Yes
12. Were the basic data adequately described?	Yes	Yes	Yes	Yes

13. Does the response rate raise concerns about non-response bias?	No	No	No	No
14. If appropriate, was information about non-responders described?	NS	NS	NS	NS
15. Were the results internally consistent?	ND	ND	ND	ND
16. Were the results for the analyses described in the methods, presented?	Yes	Yes	Yes	Yes
17. Were the authors' discussions and conclusions justified by the results?	Yes	Yes	Yes	Yes
18. Were the limitations of the study discussed?	Yes	Yes	Yes	Yes
19. Were there any funding sources or conflicts of interest that may affect the authors' interpretation of the results?	No	No	NS	NS
20. Was ethical approval or consent of participants attained?	Yes	Yes	Yes	Yes

1. Downes, M.J.; Brennan, M.L.; Williams, H.C.; Dean, R.S. Development of a critical appraisal tool to assess the quality of cross-sectional studies (AXIS). *BMJ Open* **2016**, *6*, e011458, doi:10.1136/bmjopen-2016-011458.
